# Metabolism and disposition of oseltamivir (OS) in rats, determined by immunohistochemistry with monospecific antibody for OS or its active metabolite oseltamivir carboxylate (OC): A possibility of transporters dividing the drugs’ excretion into the bile and kidney

**DOI:** 10.1002/prp2.597

**Published:** 2020-06-02

**Authors:** Kunio Fujiwara, Yutaro Yamamoto, Tetsuya Saita, Senya Matsufuji

**Affiliations:** ^1^ Department of Applied Life Science Faculty of Biotechnology and Life Science Sojo University Kumamoto Japan; ^2^ Department of Molecular Biology The Jikei University School of Medicine Tokyo Japan

**Keywords:** immunohistochemistry, Mdr4, monoclonal antibody, oseltamivir, oseltamivir carboxylate, P‐gp

## Abstract

Among any drugs, no comparative pharmacological study on how prodrug and its active metabolite behave in animal bodies is available. Immunohistochemistry (IHCs) using newly prepared two monoclonal antibodies, AOS‐96 and AOC‐160, monospecific for oseltamivir (OS) and its metabolite oseltamivir carboxylate (OC) were developed, simultaneously detecting the uptake or excretion of OS and OC in the intestine, liver, and kidney of rats to which OS was orally administered. In the intestine, IHC for OS revealed OS highly distributed to the absorptive epithelia with heavily stained cytoplasmic small granules (CSGs). IHC for OC showed that OC also distributed highly in the epithelia, but without CSGs, suggesting that OS was partly converted to OC in the cells. In the liver, OS distributed in the hepatocytes and on their bile capillaries, as well as on the lumina from the bile capillaries to the interlobular bile ducts. OC distributed in the whole cell of the hepatocytes, but without CSGs nor on any lumina through the interlobular bile ducts. In the kidney, a few levels of OS distributed in the cytoplasm of almost all the renal tubule cells, but they contained numerous CSGs. In contrast, OC distributed highly in the proximal tubules, but very slightly in the lower renal tubules of the nephrons. Thus, it was concluded that the two drugs behave in completely different ways in rat bodies. This paper also discusses a possibility of the correlation of OS or OC levels in tissue cells with their known transporters.

**Abbreviations**
AOC‐160 mAb: anti‐OC monoclonal antibody
AOS‐96 mAb: anti‐OS monoclonal antibody
BSA: bovine serum albumin
CSG: cytoplasmic small granules
ELISA: enzyme‐linked immunosorbent assay
GA: glutaraldehyde
IEM: immunoelectron microscopy
IHC: immunohistochemistry
Mdr1: multidrug resistance protein 1 (P‐glycoprotein, P‐gp)
Mrp4: multidrug resistance‐associated protein 4
OC: Oseltamivir carboxylate
OS: Oseltamivir;
TBS: 50 mM Tris‐HCl buffer, pH 7.4, containing 0.86% NaCl
TBST: TBS supplemented with 0.1% Triton X‐100

## 1 INTRODUCTION

Oseltamivir (OS) is an ethyl ester‐type pro‐drug of Ro 64‐0802 (oseltamivir carboxylate; OC), a potent and selective inhibitor of viral neuraminidase, a key enzyme involved in the release of the influenza virus from infected host cells.^1^, ^2^ OS is used for the prevention, treatment, and prophylaxis of epidemic seasonal influenza of Influenza virus A and B.^3^ An ethyl ester group of OS is enzymatically cleaved to the active metabolite OC predominantly in the plasma in rats,^4^ while in humans (and monkeys), the cleavage takes place in the liver,^5^ and both OS and OC are found in the blood.^6^, ^7^ The accurate localization of OS and OC in tissue cells would help to develop better understanding of the biological response to the drugs in the pharmacological studies. Previous distribution studies for drugs in animals have been almost exclusively undertaken by autoradiography, as reported by Wiltshire et al^8^ using 14C‐labeled OS, but without detailed information. We searched for an alternative procedure for immunohistochemistry (IHC), which has the advantage over autoradiography of being able to directly observe the tissue localization of drugs using a common light microscope. Also, in principle, IHC detects a drug molecule itself according to the specificity of the antibody (Ab) and is as highly sensitive of the assay procedure as autoradiography. Furthermore, it does not need any special skills during the operation and can be completed within less than a couple of days. However, there were no reports available on IHC for drugs developed by pharmacologists or histologists until 2005.^9^ Prior to our starting to investigate IHC for drugs, we had been engaged in the research for low molecular weight endogenous amines, polyamines,^10^ and histamine,^11^ by IHC procedure, in which Abs specific for their amines were prepared and used with light and electron microscopy. Our studies revealed that polyamines localized on the free and attached ribosomes in any cell types,^12^ and also that histamine in rats is concentrated in the specific cores of cytoplasmic granules in the enterochromaffin‐like cells of the stomach.^13^ Those studies taught us that for developing the IHC for small molecule compounds, it was critically important to set up the accurate conditions of the fixation, especially for the small water‐soluble antigens in situ*,* ^12^, ^13^ and also understand how to prepare Ab,^14^, ^15^ the antigen masking effect caused by the fixative,^16^ its unmasking procedure for reaction with Ab(s),^10^, ^11^, ^16^ and how to remove the non‐specific binding of Ab.^17^ We, therefore, adapted these understandings to the development of IHCs for drugs especially with primary aliphatic amino group(s) in their molecules.^9^ In addition, small molecular endogenous amines have been extensively studied with IHCs by others.^18^ The pharmacodynamics of pro‐drug together with its active metabolite at the cell level would be valuable in the animal bodies because no such knowledge so far exists. Both OS and OC are ideal drug molecule models with an aliphatic amino group as the fixation site in situ for IHC because a simultaneous comparative study by autoradiography might be almost impossible to perform. Also, as the transport systems responsible for OS or OC uptake or excretion a member of the ATP binding cassette (ABC) transport proteins Mdr1 (P‐glycoprotein: P‐gp),^19^, ^20^, ^21^, ^22^, ^23^ the organic anion transporter (OAT1),^24^ the OAT3,^1^ multidrug resistance‐associated protein 4 (Mrp4/abcc4),^25^, ^26^, ^27^ and the peptide transporter1 (Pept1)^28^, ^29^, ^30^, ^31^ have been demonstrated by pharmacological studies using knockout mice or cell lines expressing such transporters. However, the in vivo role of transporters in drug disposition, and metabolism, has been scarcely established. We have tried a few studies on the correlation of transporters with drug disposition,^32^, ^33^ not yet obtaining enough information on it, because there are no examples of drugs comparable for each other.

We now report on the development of two mAbs monospecific for OS and OC, and the two IHCs for the uptake of OS and OC in the small intestine, liver, and kidneys of rats that were administered OS orally. The finding that both OS and OC which were uptaken into the hepatocytes flow into the bile capillary and the sinusoidal capillary possibly through the P‐gp and Mrp4, respectively, suggests that the transporter(s) play a critical role in dividing the excretion of both of the drugs into the bile and kidney. Also, it is suggested that OS partly undergoes the first‐pass hydrolysis into OC within the intestinal epithelia, and that OS, but not OC, might be classified into the “Lysosomotropic" cationic amphiphilic drug category. The IHC clearly demonstrated its utility as a new tool for pharmacology and toxicity studies at the cell level.

## 2 MATERIALS AND METHODS

### 2.1 Chemicals

Tamiflu was from Chugai Pharmaceutical, Tokyo, Japan. Glutaraldehyde (GA, 25% in water) was obtained from Nacalai Tesuque. Protease (Type XXIV: Bacterial) was from Sigma‐Aldrich. Co. Inc.

### 2.2 Oseltamivir (OS) and Oseltamivir carboxylate (OC)

OS was extracted with ethyl acetate from Tamiflu^™^ dissolved in 10 mmol/L borate buffer, pH 10, and then treated with saturated NaCl, and confirmed by FAB^+^ Mili Mass spectrum *m*/*z* 313.3 (M + H)^+^ (C_16_H_29_N_2_O_4_), and also by HPLC equipped with a LiChroCART®125‐4i.d. cartridge [RP‐C18ODS(e) Lichrosphere 100] (Merk) with a mobile phase of acetonitrile: 10 mM NaH_2_PO_4_ containing 0.1% TFA (60:40) with a single peak of the retention time of 1.6 minutes (flow rate, 1.0 mL/min). OC: OS was incubated with 0.1 mol/L NaOH for 1 hour at room temperature, and the hydrolyzed compound OC was confirmed to be homogenous by the HPLC with the retention time of 1.2 minutes and used for specificity of the mAb by the inhibition and binding ELISAs described below.

### 2.3 Preparation of OS‐ or OC‐bovine serum albumin (BSA) conjugates

OS‐GA‐BSA conjugate was prepared according to our previous method using GA as cross‐linking agents.^10^ OS (approx. 30 mg) was dissolved in 2.0 mL of 0.12 mol/L borate buffer, pH 10, and 15 µg of GA was mixed and incubated at room temperature (RT) for 30 seconds with stirring, and to the mixture was then added BSA (15 mg) in 1.0 mL of the buffer. This was followed by incubation for 30 minutes before NaBH_4_ (5 mg) was added to terminate the reaction. The reaction mixture was further incubated for 30 minutes with slow stirring. The conjugate was then purified by a column chromatography of Sephadex G‐75 equilibrated with 10 mmol/L phosphate buffer, pH 7.0 containing 4 mol/L urea. Also, OC‐GA‐BSA was prepared in the same manner as described above using OC instead of OS. The resulted conjugates OS‐GA‐BSA and OC‐GA‐BSA were used as immunization antigens for anti‐oseltamivir (AOS) and anti‐oseltamivir carboxylate (AOC) monoclonal antibodies (mAbs), respectively, or also as solid‐phase antigens or inhibitors for an enzyme‐linked immunosorbent assay (ELISA) described below.

### 2.4 Preparation of AOS and AOC mAbs

As for the AOS mAb, three five‐week‐old, female BALB/c mice were injected intraperitoneally (i.p.) with 100 µg of OS‐GA‐BSA conjugate emulsified in complete Freund's adjuvant (Difco). Subsequently, they received three injections of 50 µg of the conjugate alone at two‐week intervals. Following immunization, antisera were collected, and antibody titers were evaluated with ELISA as described below. The mouse with the best immune response was selected for hybridization. The mouse received a fourth i.p. booster injection and was sacrificed 4 days later. Experiments for the AOC mAb were similarly carried out using the conjugate OC‐GA‐BSA as the immunogen.

### 2.5 Cell fusion and cloning

In these experiments for either AOS or AOC, the spleen cells (2 × 10^8^) from the immunized mouse and 3x10^7^ myeloma cells (P3/NS‐1) were fused with the help of polyethylene glycol according to our previous method.^10^ Cells suspended in HAT medium were plated out in 96‐well tissue culture plates (Corning) at a density of 10^5^ cells per well in which 10^5^ feeder cells had been plated. From 10 to 20 days postfusion, the wells were screened for reactivity using an ELISA method, as described below. Limiting dilutions of positive cultures were carried out two or three times to obtain monoclonality, and sub‐isotyping of the mAbs was performed using a Mouse Monoclonal Sub‐isotyping kit (American Qualex Int.). Ascites were raised in BALB/c mice pretreated with Pristene by intraperitoneal injection of 2 × 10^6^ hybridoma cells.

### 2.6 Dilution ELISA

ELISA was performed similarly to our previous method for anti‐spermine mAbs.^10^ In screening clones for production of antibody against OS‐GA‐BSA (or OC‐GA‐BSA), wells in microtiter plates were coated with 10 µg/mL of each of the conjugates for 30 minutes at RT. The wells were then incubated overnight at 4°C with antiserum (diluted 1:3000), hybridoma culture supernatant, or ascites fluid (diluted 1:100 000), followed by goat anti‐mouse IgG labeled with horseradish peroxidase (HRP) (diluted 1:2000) for 1 hour at 25°C. The amount of enzyme conjugate bound to each well was measured using o‐phenylenediamine as a substrate, and the absorbance at 492 nm was read with an automatic ELISA analyzer (ImmunoMini NJ‐2300, Nalje Nunc Int. Co. Ltd.).

### 2.7 Inhibition ELISAs^34^, ^35^

Wells in a microtiter plate are coated with 100 µL of the OS‐GA‐BSA (10 µg/mL) (or OC‐GA‐BSA) as described above. To the coated wells were added 50 µL of a fixed concentration of AOS‐96 mAb (1:100), (or AOC‐160 mAb) and then 50 µL of different conjugates (OS‐GA‐BSA, OC‐GA‐BSA) or free compounds (OS, OC, daunomycin, and mitomycin C) at various concentrations, and these were incubated overnight at 4°C, followed by incubation with goat anti‐mouse IgG labeled with HRP (1:2000) for 1 hour at RT. The bound HRP activity was measured using the ELISA analyzer.

### 2.8 Binding ELISAs

According to our previous method,^10^ the wells in a microtiter plate coated with poly‐L‐lysine (30 µg/mL) were activated with 2.5% GA in 50 mmol/L borate buffer, pH 10.0, for 1 hour The wells were subsequently incubated with test compounds at various concentrations for 1 hour at RT. Excess aldehyde groups were blocked with 0.5% sodium borohydride. The wells were further incubated for 1 hour with 1% skimmed milk to block nonspecific protein binding sites, and then the plates were incubated overnight at 4°C with the primary AOS‐96 (or AOC‐160) at 1:50 diluted with phosphate‐buffered saline (PBS) containing 0.05% Tween 20 (PBST). The wells were then incubated for 1 hour with HRP‐labeled goat anti‐mouse IgG (diluted 1:2000). The bound enzyme activity was measured as described above.

### 2.9 Animals

Normal adult male Wistar rats (Kyudo Exp. Animals), body weight 200‐250 g, were used in this study. The principles of laboratory animal care and specific national laws were observed. The animals were housed in temperature‐ and light‐controlled rooms (21 ± 1°C and 12L: 12D) and starved overnight but with free access to tap water. Single oral doses of 20 mg OS/kg of body weight were administered to nine Wistar rats. At 1, 3, 12, and 24 hours after the administration three rats for each group were anesthetized and perfused transcardially with 2.0% GA in PBS (10 mmol/L phosphate buffer, pH 7.2, containing 0.15 mol/L NaCl). The small intestine, liver and kidney were quickly excised and postfixed in the same fixative GA overnight at 4°C and was subsequently routinely embedded in paraffin.

### 2.10 Immunohistochemistry (IHC)

The IHC method for OS or OC was carried out essentially according to our previous methods.^33^, ^36^, ^37^ While they were under sodium pentobarbital (60 mg/kg; Abbott Laboratories) anesthesia, the rats were perfused intracardially with PBS at 50 mL/min for 2 minutes at RT and then with a freshly prepared solution of 2% GA in 10 mmol/L phosphate buffer, pH 7.2, for 6 minutes. Kidney, liver, and intestine (the duodenum) were quickly excised and postfixed in the same fixative overnight at 4°C and were subsequently embedded in paraffin in a routine way. The samples were cut into 5 µm thick sections, digested with 0.003% protease (type XXIV, bacterial; Sigma‐Aldrich) in 50 mmol/L Tris‐HCl buffer, pH 7.4, containing 0.86% NaCl (Tris‐buffered saline [TBS]) for different periods (15 minutes to 2 hours) at RT (to facilitate the penetration of antibody to fully detect uptake of OS or OC in different cells and subcellular compartments), and reduced with 1.0% NaBH_4_ in TBS for 10 minutes (to prevent unspecific staining by reducing free aldehyde groups of GA bound in tissues). During each process of the treatment, the specimens were washed three times with TBS containing 0.9% sodium metabisulphite (SMB). Next, the specimens were blocked with a protein solution containing 10% normal goat serum (NGS) supplemented with 0.1% NaN_3_, 1.0% BSA, 0.9% SMB, and 0.1% saponin in TBS for 1 hour at RT and were then directly incubated at 4°C overnight with either AOS 96 or AOC 160 mAb diluted 1:20 in TBS supplemented with 0.1% Triton X‐100 (TBST). The sections were washed with TBST three times for 5 minutes each time and then incubated with N‐Histofine® Simple Stain MAX‐PO (Multi) (Universal Immuno‐peroxidase Polymer, Anti‐mouse and –Rabbit; Nichirei BioScience Inc Co. Ltd.) diluted 1:2 in TBS supplemented with 0.1% saponin, 1% NGS without NaN_3_, and 0.25% BSA in 50 mmol/L TBS for 120 minutes at RT. After the sections were rinsed with TBS, the site of the antigen‐antibody reaction was revealed for 10 minutes with diaminobenzidine (DAB) and H_2_O_2_.

In the present studies, the optimal conditions for immunostaining with respect to protease digestion differed in the different cell tissues. These differences may reflect differences in the binding and masking of OS or OC by proteins and other macromolecules in different cellular compartments, which may thus constitute different obstacles to the penetration of the antibody into such cell tissues. In this study, the three different IHC procedures were mainly conducted by changing the periods for the protease (0.003%) digestion: namely, IHCs with no protease digestion (IHC‐N), with mild protease digestion for 15‐30 minutes (IHC‐M), and with strong protease digestion for 2 hours (IHC‐S), in the conditions of which the former one detects non‐masked drug and the latter two detect masked drug.^38^, ^39^ Importantly, unmasking of tissue cell specimens by the protease digestion are commonly essential, especially in the IHC when GA or Karnovsky fixative (1965)^40^ etc is used as a fixative for covalently fixing in situ the mobile small molecular compounds such as the endogenous amines, polyamines,^41^ histamine,^11^ serotonin,^42^ dopamine,^43^ GABA,^44^ glutamic acid,^45^ and drugs,^46^ since these molecules are differently fixed in situ within cells via their amino group which reacts with the one of the two aldehyde groups of the bifunctional cross‐linker GA, forming Schiff base (C = N). The other aldehyde group at the other end of the GA may be also bound in the same way with an amino group of some protein(s) etc, present in situ. Then, the two Schiff bases are reduced with NaBH_4_, giving the chemically stable C‐N bonds, as described above. Antibody (Ab) a high molecular immunoglobulin (IgG) cannot come through the membrane into the cytoplasm, nuclei, mitochondria, and lysosomes etc within cells in tissues, unless the cell specimens are pre‐treated with protease,^38^ with the exception of an antigen drug on or near the cell surface. In contrast to this, we have previously observed that in drug IHC‐N significant immunostaining occasionally occurred over the whole cell and the cells got abnormally large or swollen.^36^ Perhaps the cells were affected or broken down by the excessive uptake of the drug. Accordingly, Ab freely comes through within the cells and tissues. In addition, it is generally known that in IHC the stronger the fixation is, the more strongly the protease excavation for unmasking antigen is ^10^, ^11^, ^16^. In these cases, however, the cell morphology gets poorer due to the disruption of the cell structures, even to the point of cases where the superficial antigen drug nearly completely washed away. Also, the smaller the Ab (particularly, the secondary Ab) is made, for example, F(ab’)2, Fab’, or Fab etc, the better it enters cells.^47^

The present IHCs for OS or for OC had characteristic advantages of using the tissue sections from the same paraffin block, and therefore would give this comparative study for drugs’ tissue localization more accurate data, because it is free from experimental errors which may occur from the use of different animals and the effects of differing skills in the transcardial fixation procedures.

Control experiments: In the IHC for OS or OC in the small intestine, liver and kidney of rats to which OS was administered, the specificity of immunostaining was ascertained by incubating sections with the secondary antiserum alone (the secondary level controls), and AOS‐96 mAb and AOC‐160 mAb preabsorbed with OS‐GA‐BSA (100 µg/mL) and OC‐GA‐BSA conjugate (100 µg/mL), respectively.

### 2.11 Immunoelectron microscopy (IEM)

When immune‐positive reactions occur in the light microscopic studies of IHC‐N, it is the sign of a possibility of the development of IEM due to preservation of the intact ultrastructure, being free from protease digestion.^12^, ^13^, ^46^ The post‐fixed specimens of the liver were cut into 50‐μm‐thick sections with a Microslicer (Dosaka EM; Kyoto, Japan), which were then applied to a free‐floating procedure of the pre‐embedding method,^12^, ^13^, ^46^ in principally the same manner as was used for ICC for paraffin sections, except for the HRP‐labeled goat anti‐mouse IgG/Fab’ (MBL; Nagoya, Japan) used as the secondary antibody. The color‐developed specimens obtained were then fixed with 1.0% osmium tetraoxide in 50 mmol/L cacodylate buffer, pH 7.4, for 1 hour at RT and dehydrated in a series of graded ethanol solutions. After immersion in propylene oxide (Nakalai Tesque) (three times for ten min each), the samples were immersed in a mixture (1:1) of propylene oxide and Epon 812 resin (Taab Lab; reading Berks, UK) overnight and embedded in Epon 812 resin in a routine way. The regions to be studied were cut with a 2‐mm diameter punch, mounted to Epon blocks, and sectioned into ultrathin sections (LKB 8800 Ultrotome^®^III), which were then immediately observed in a 100CX Jeol electron microscope.

## 3 RESULTS

Generation and detection of mAb to OS or OC: From a fusion experiment for AOS 420 hybridoma lines were produced. Five hybridoma lines (AOS‐34, 64, 72, 96, and 99) secreted antibodies that bound to the OS‐GA‐BSA conjugate but did not recognize BSA, as determined by ELISA. They continued to secrete antibodies in culture supernatant. Subclones of the hybridoma obtained by limiting dilution of AOS‐96 was found to produce an antibody of the IgG type, but its sub‐isotype was not determined, since no reaction occurred with any of the IgG subtype‐antibodies available in Mouse MonoAb‐ID kit (Zymed Lab. Inc). The others 4 AOS were of all the IgM sub‐isotype. As regards preparation of the AOC, three hybridoma lines (AOC‐15, 88, and 160) among 445 hybridoma lines were produced binding to the OC‐GA‐BSA conjugate. The AOC 160 line secreted and continued the IgG1 sub‐isotype mAb, and the other two were of the IgM sub‐isotype.

**FIGURE 1:**
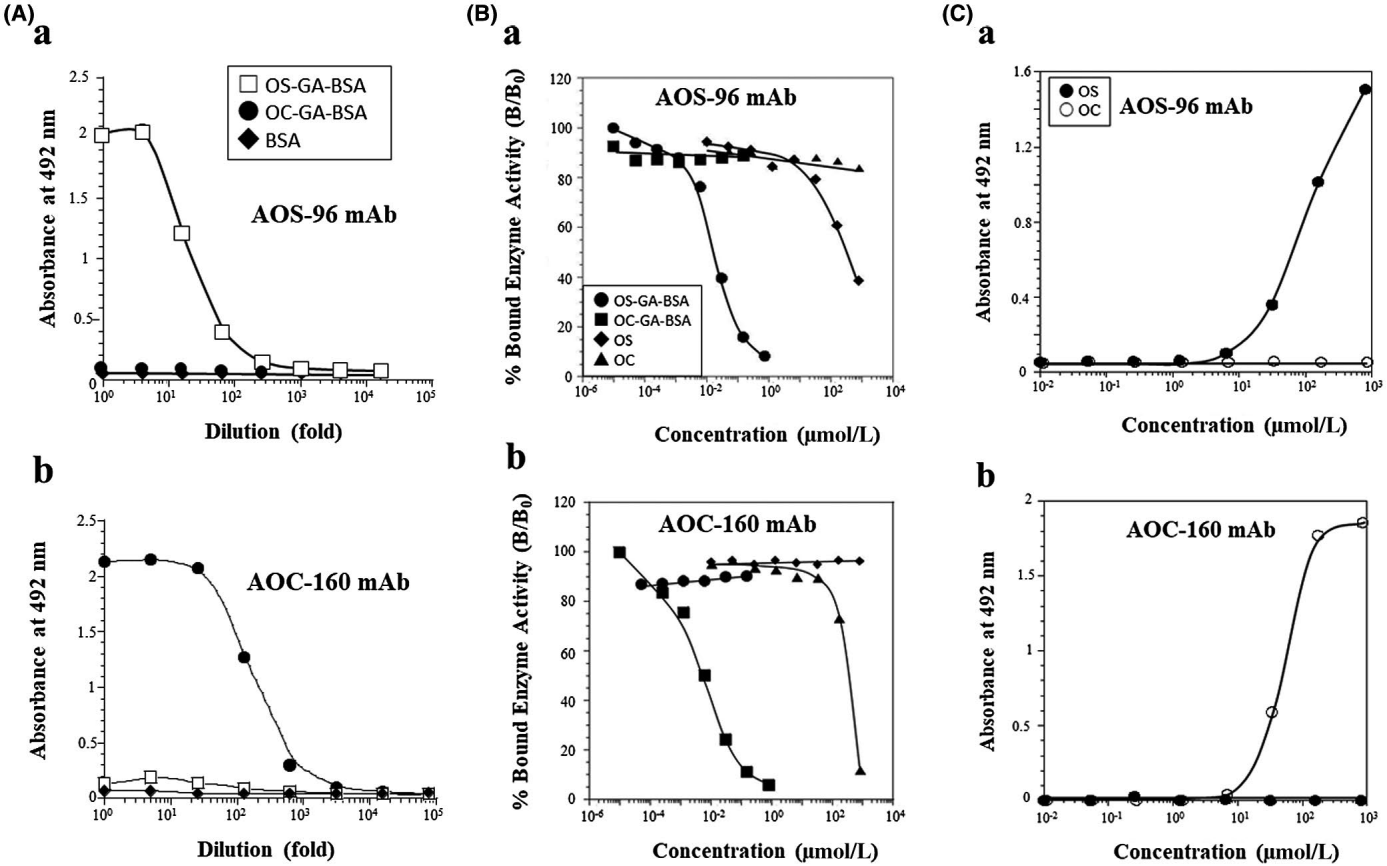
(A) ELISA measurements of the binding of serially diluted anti‐OS or OC monoclonal antibody [AOS‐96 (a) or AOC (b)] to the solid phase coated with the conjugate of OS‐GA‐BSA or OC‐GA‐BSA, or BSA. (B) Reactivity of the mAb AOS‐96 (a) or AOC‐160 (b) as measured by its immunoreactivity in the inhibition ELISAs. The curves show the amount (percentage) of bound enzyme activity (B) for various doses of OS‐GA‐BSA, OC‐GA‐GA‐BSA, or free OS or OC as a ratio of that bound using the HRP‐labeled secondary antibody alone (B_0_). The concentration of OS or OC in the conjugate OS‐GA‐BSA or OC‐GA‐BSA, was photometrically calculated, assuming the molar extinction coefficient of BSA to be 43 000 at 280 nm, and also that of the other part (OS‐, OC‐, OS‐GA‐, or OC‐GA) of each the conjugates to be ignored. (C) Reactivity of the mAb AOS‐96 (a) or AOC‐160 (b) determined from its immunoreactivity in the binding ELISA. Activated wells prepared for the binding ELISA were incubated with various concentrations of OS or OC. The wells were reacted with NaBH_4_ and then with HRP‐labeled goat anti‐mouse IgG (whole; 1:2000)

Antibody dilution: Microtiter wells coated with the conjugate either OS‐GA‐BSA or OC‐GA‐BSA (each 10 µg/mL) were used to test for antibody binding (using serial dilutions) for the mAb of AOS‐96 or AOC‐160 (hybridoma culture supernatants). The AOS‐96 mAb showed significant binding activities for OS‐GA‐BSA conjugate in dilutions of less than 1:100, but none for OC‐GA‐BSA conjugate even in much lower dilutions (Figure 1Aa). On the contrary, mAb AOC‐160 showed high binding activities for OC‐GA‐BSA conjugate, but none for OS‐GA‐BSA conjugate in any dilutions (Figure 1Ab). No antibody binding was seen in any of the conjugates with IgG1 mAb of ASPM‐29, known to be specific to polyamines, spermine, and spermidine (Fujiwara et al 1995) (data not shown).

Inhibition ELISA for the AOS mAb or for the AOC mAb: This was achieved by the principle of competition between OS, OC, OS‐GA‐BSA, or OC‐GA‐BSA (free in solution), and a fixed amount of OS‐GA‐BSA coated on ELISA plates for the limited number of binding sites on the AOS‐96. Calibration curves were plotted showing the relationship between the concentrations of the analytes and the percentage of bound mAb, giving dose‐dependent inhibition curves with the conjugate OS‐GA‐BSA in the range between 1 nmol/L and 100 µmol/L (Figure 1Ba). The dose required for 50% inhibition of binding was used as an indication of the strength of inhibition. This dose (EC50) was 5.7 nmol/L with OS‐GA‐BSA and 340 nmol/L with free OS. No inhibition occurred with OC‐GA‐BSA conjugate, OC, and other antibiotics, daunomycin, and mitomycin C tested even at concentrations of less than 1 µmol/L (data not shown). Inhibition ELISA for the AOC‐160 mAb: This was similarly performed using the OC‐GA‐BSA conjugate as the solid phase antigen. The EC50 dose was 5.7 nmol/L with OC‐GA‐BSA and 350 nmol/L with free OC. In contrast, no reaction occurred with OS‐GA‐BSA, OS and other drugs (Figure 1Bb).

Binding ELISA for the AOS mAb or for the AOC mAb: The binding ELISA simulates the ICC of tissue sections, based on the principle of coupling of the amino group of the analytes to the wells of a microtiter plate activated with poly‐L‐lysine and GA and incubation of the wells by the indirect immunoperoxidase method.^48^ As shown in Figure 1Ca, analysis of the relationship between the concentration of each of the analytes applied to the wells and the bound HRP activity produced a dose‐dependent curve, with OS concentrations ranging from 10 µmol/L to 1 mmol/L as the amounts incubated in the wells. No reaction with OC, amoxicillin or gentamicin occurred. Binding ELISA for the AOC mAb: Immunoreaction occurred only with OC ranging from 10 µmol/L to 1 mmol/L in dose‐dependent manner in the tested compounds, sharply in contrast to that for the AOS (Figure 1C and Cb).

### 3.1 Drug uptake in intestine

**FIGURE 2:**
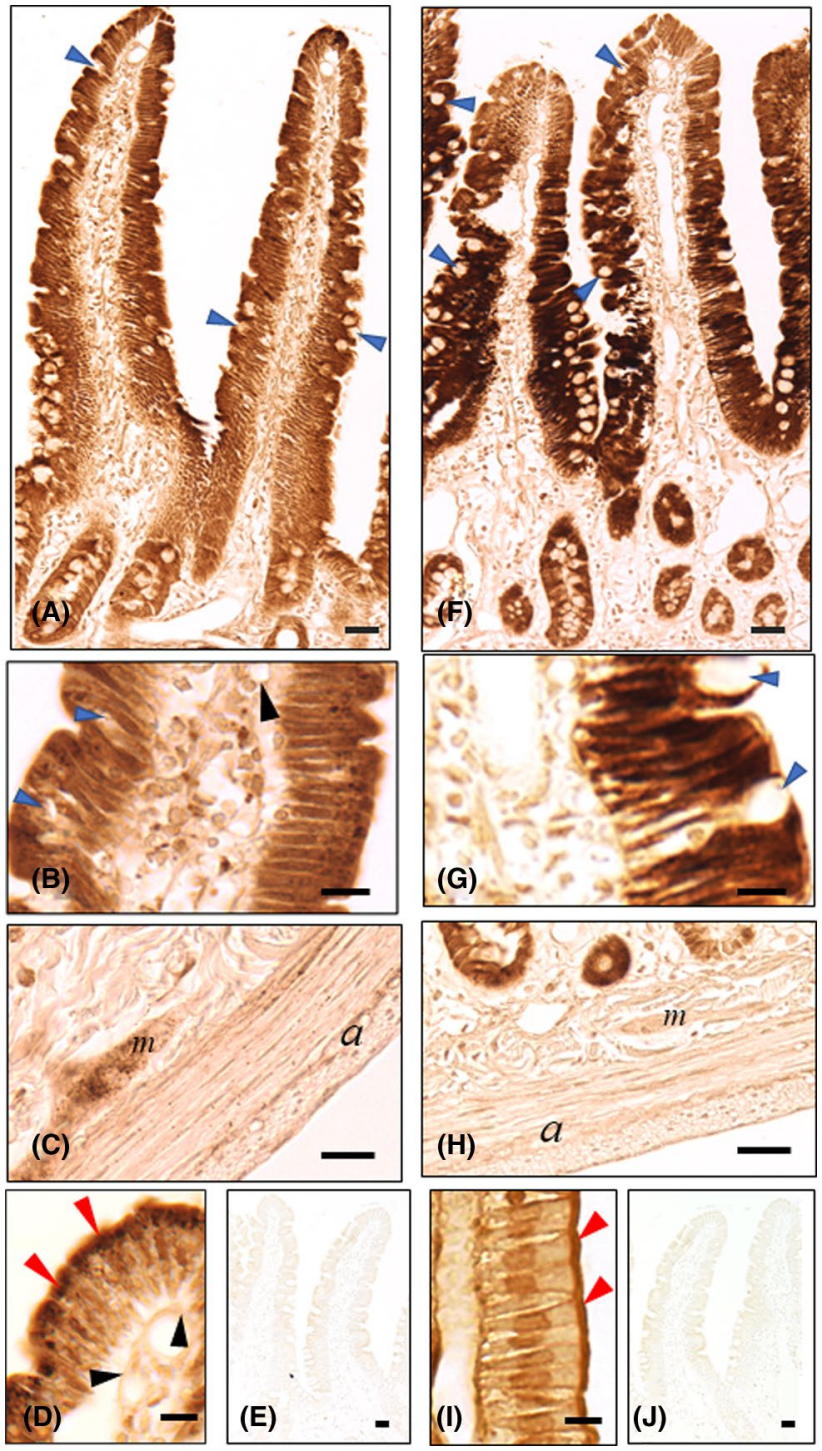
(A‐I) Immunostaining for OS or OC in small intestine of OS‐administered rats. Rats were orally administered OS at 20 mg/kg, and then sacrificed 3 hours later. Five µm paraffin sections from small intestine block from OS‐administered rats were prepared. Then, the section specimens were digested with protease at 0.003% at 30°C either for 30 minutes (D, I IHC‐M) or for 2 hour (A‐C, E, F‐H, J IHC‐S), followed by the first immunoreaction either with AOS‐96 mAb (A‐E) or with AOC‐160 mAb (F‐J). A, F Duodenum (lower magnification): Immunostaining either for OS (A) or OC (F) strongly occurred in the absorptive epithelial cells, less strongly in the crypt cells, but almost none in the goblet cells (blue arrowheads). (B) (Higher magnification): Strong immunoreaction for OS was observed in the numerous cytoplasmic small granules (CSGs) in the upper half of the cytoplasm of the absorptive epithelial cells. (G) No CSG was observed anywhere in the absorptive epithelial cells. (B, G) Some wondering cells (the free cells: lymphocytes, macrophages, plasma cells etc) were more strongly stained for OS (B) than for OC (G) in the lamina propria mucosae. (C) The CSGs were observed in both the plexus cells of Meissner and Auerbach and around in the smooth muscle cells. H Contrast to Figure 2C, IHC‐S for OC produced no CSGs in their respective area. D, I Strong staining for OS (D) or for OC (I) was pronounced in the microvilli (red arrowheads) of the absorptive epithelial cells, although in the IHC‐S for OS or OC almost all the microvilli of the epithelium cells were washed away during the processes of the protease digestion for 2 hours (A‐C, E; F‐H, J) and the subsequent washings with TBST. E and J Staining for OS and for OC were completely abolished by absorption of AOS‐96 mAb with OS‐GA‐BSA (100 µg/mL) (E) and of AOC‐160 mAb with OC‐GA‐BSA (100 µg/mL) (J), respectively. *a* Auerbach's plexus; *m* Meissner's plexus; *Bars* A, E, F, J = 20 µm; B, C, D, G, H, I = 10 µm

Three hours after a single oral dose of OS at 20 mg/kg, the IHC‐S for OS using the AOS‐96 mAb (the culture supernatant) produced strong staining for OS in the brush border microvilli (Figure 2D), nuclei and cytoplasm of the absorptive epithelial cells in the specific morphology of the cytoplasmic small granules (CSGs) in the upper half of the cells, strongly reacting with the mAb (Figure 2A‐D). Also, of note is that such CSGs occurred all over the other cell types in the intestine described below. Less strong or moderate staining occurred in the whole cell of the intestinal gland cells of Lieberkuhn and both in the cytoplasm of the plexus cells of Meissner and Auerbach (Figure 2C) as well as in the wondering cells (the free cells: lymphocytes, macrophages, plasma cells etc) (Figure 2B) in the lamina propria. Weak staining was seen in the endothelial cells (Figure 2B and D) and in the periphery of the nuclei of the smooth muscle cells (Figure 2C). No or only very slight staining was observed in the mucin goblets of the epithelium (Figure 2A and B) and in the nuclei of the two types of plexus cells (Figure 2C). One h post administration, the IHC‐S for OS showed stronger staining in the intestine but their patterns were almost the same as what was obtained at 3 hour post administration. Twenty‐four h post administration, no staining almost in any cell types, but some staining persisted especially in the cells of the upper half the absorptive epithelium (data not shown).

The IHC‐S for OC was carried out in the same manner as IHC‐S for OS, except using the AOC‐160 mAb. Comparison of the staining for OC to that for OS was made using AOC mAb equally diluted to the AOS mAb (1:20), because each of the mAbs showed almost the same sensitivity to the drug, as shown in the inhibition ELISA tests (EC50 dose: 340 µmol/L for OS and 350 µmol/L for OC) (Figure 1Ba and B): Importantly, very strong staining for OC in all three parts of the microvilli, cytoplasm, and nuclei of the epithelium was observed similarly to that for OS (Figure 2F, G and I). However, there were differences in that first, there were no more CSGs stained for OC in any cell types of the intestine (Figure 2F, G and H), second no or only very slight staining for OC was found both in the plexus cells of Meissner and Auerbach (Figure 2H), and third, the free cells in the lamina propria were less weakly stained for OC than for OS (Figure 2F and G), although the epithelium cells seemed to be rather more strongly stained for OC than for OS (Compare Figure 2F and G to A and B). Staining tendency with IHCs for OC at 1 and 24 hour post administration was almost similar to that with IHCs for OS at 1 and 24 hour post administration, respectively, but without CSGs in any cell types (data not shown). The controls, including the secondary level controls (data not shown) and the absorption controls, in which OS‐GA‐BSA conjugate (100 µg/mL) or OC‐GA‐BSA conjugate (100 µg/mL) were added to the first immunoreaction of the intestine specimens with either AOS‐96 mAb or the AOC‐160 mAb, were all negative (Figure 2E and J).

### 3.2 Drug uptake in liver

**FIGURE 3:**
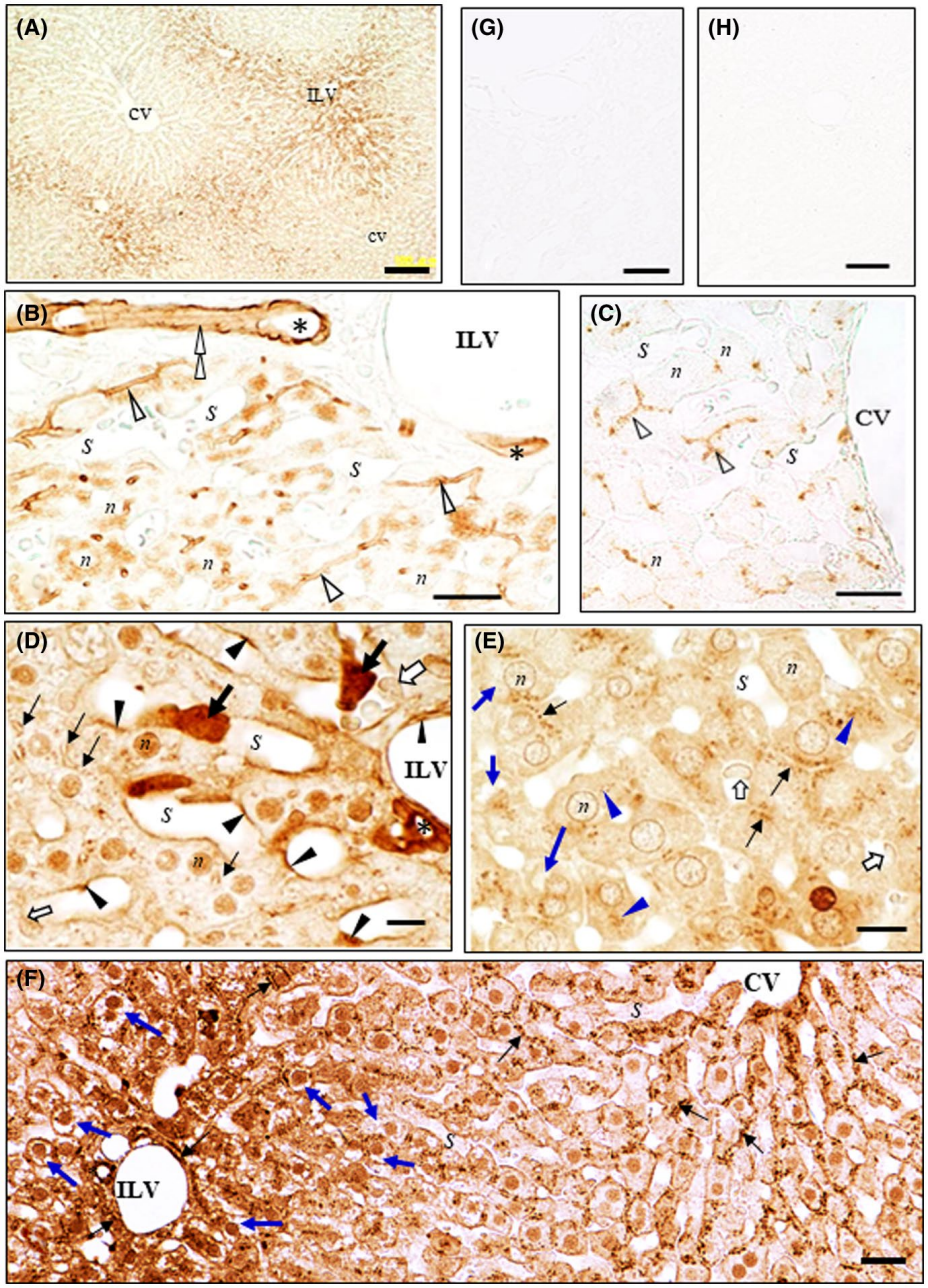
(A‐H) Immunostaining for OS in the liver of OS‐administered rats. Rats were orally administered OS at 20 mg/kg and then sacrificed one h later. IHC for OS was carried out without protease digestion (A‐C IHC‐N) or following digestion of sections with protease at 0.003% at 30°C for 30 minutes (D IHC‐M) or 2 hour (E, F IHC‐S) prior to immunoreaction with AOS‐96 mAb. (A) Lower magnification: Staining was observed in the hepatocytes around the interlobular veins (ILV) but not in ones around the central veins (CV). (B and C) Higher magnification: Immunoreaction occurred in the specific parts of the cytoplasm peripheral to the nuclei of the hepatocytes and in the luminal surface of the bile capillaries (open arrowheads), intercalated portion (open double arrowheads), and interlobular bile duct cells (asterisks). (D) Immunoreaction ranges from very strong staining with the larger cells (thick black arrows) in the sinusoids and in the interlobular bile duct cells (asterisks), moderate immunostaining in the nuclei (*n*) of the hepatocytes and the endothelial cells (black arrowheads) to weak staining in the Kupffer cells (open arrows). Of note is that the numerous small spots seem to be appearing between the hepatocytes (thin black arrows). (E and F) Numerous small spots (thin black arrows) appeared to line along to the bile capillaries between the hepatocytes, together with a very few CSGs (blue arrowheads). Kupffer cells were also weakly stained for OS (open arrows). The endothelial cells were no longer observed, since they were hydrolyzed and washed away in the protease digestion process of IHC‐S for OS. (F) Lower magnification: Differences in immunostaining for OS in hepatocytes around the interlobular vein (ILV) and the central vein (CV). Strong immunostaining of numerous small spots is observed among hepatocytes everywhere in the liver. Immunostaining in the nuclei and cytoplasm of the hepatocytes around ILV was stronger than those around CV, while colorless gaps were found only in the cytoplasm of the hepatocytes around ILV, possibly due to their damage by the protease digestion process in the IHC‐S for OS. (G) Staining was completely abolished by absorption of the AOS‐96 mAb with OS‐GA‐BSA (100 µg/mL). (H) No staining in the liver from control rats. CV central vein; ILV interlobular vein; *S* sinusoids; *n* nuclei; *m* mitochondria; * interlobular bile ducts. *Bars* A, F = 100 µm; B, C = 20 µm; D, E = 10 µm; G, H = 50 µm

**FIGURE 4:**
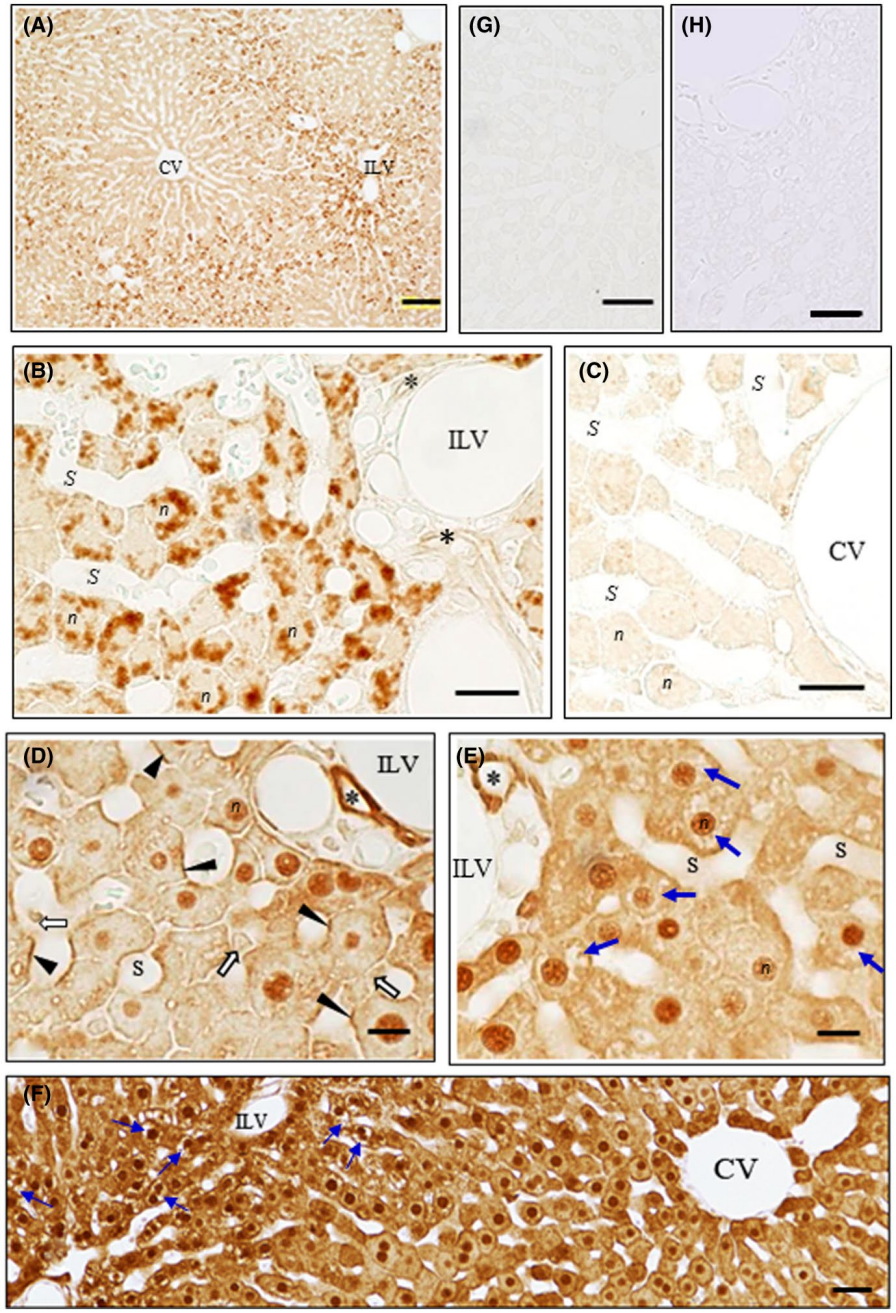
(A‐H) Immunostaining for OC in the liver of OS‐administered rats. The paraffin blocks were the same as used for the immunostaining for OS by IHC, but AOC‐160 mAb instead of AOS‐96 mAb was employed. IHC was carried out either without the protease digestion process (A‐C IHC‐N) or following digestion of sections with protease at 0.003% at 30°C for 30 minutes (D IHC‐M) or 2 hour (E‐H IHC‐S) prior to immunoreaction with AOC‐160 mAb. (A) Lower magnification: Staining was strong in the hepatocytes around the interlobular veins (ILV), in contrast to only very weak in the hepatocytes around the central veins (CV), whose staining pattern is quite similar to that of Figure 3A. (B) Higher magnification: Immunoreaction was observed only in the specific parts of the cytoplasm peripheral to the nuclei of the hepatocytes, but not at all in the luminal surface of the bile capillaries and intercalated portion. Under this IHC‐N condition very faint immunoreaction for OC was also seen in the interlobular bile duct cells (asterisks). (C) No or only very faint immunoreaction was observed in the nuclei and cytoplasm of the hepatocytes around the central vein. (D) Immunostaining varies from strong with nuclei (*n*) of hepatocytes, with the interlobular bile duct cells (asterisks), and with the endothelial cells (black arrowheads), moderate staining with the cytoplasm near the plasma membranes of hepatocytes to weak with Kupffer cells (open arrows). (E) No or very faint immunoreaction was observed in the cytoplasm peripheral to the nuclei of hepatocytes around the interlobular vein (blue arrows). (F) In striking contrast to IHCs‐S for OS (Figure 3E, F), neither small spots nor CSGs occurred in any hepatocytes all over the liver. Staining intensity in the hepatocytes around the interlobular vein (ILV) was almost the same as that around the central vein (CV), while colorless gaps were observed only in the cytoplasm of the hepatocytes around ILV, as those in IHC‐S for OS (Figure 3F). (G) Staining was completely abolished by absorption of the AOC‐160 mAb with OC‐GA‐BSA (100 µg/mL). (H) No staining in the liver of the control rats. CV central vein; ILV interlobular vein; *S* sinusoids; *n* nuclei; *m* mitochondria; * interlobular bile duct. *Bars* A, F = 100 µm; B, C = 20 µm; D, E = 10 µm; G, H = 50 µm

In the liver at 1‐h post administration, in the lower magnification, the **IHCs‐N for OS or for OC** produced the significant levels of each drug was observed in the specific parts of the cytoplasm peripheral to the nuclei of the hepatocytes around the Glisson's capsules (Figures 3A‐C and 4A‐C). In contrast, by **the IHC‐S for OS or for OC** almost no immunostaining occurred in such specific parts of the cytoplasm (Figures 3E, F and 4E and F). Also, the parts seem to get morphologically somewhat wounded, possibly due to the protease digestion in the IHC (Figures 3E and 4E). Thus, cell morphology of the hepatocytes around the Glisson's capsules was compared with ones around the central veins by the HE staining, OS‐ or OC staining using the protease‐treated or nontreated specimens of the liver from OS‐administered rats or controls (Figures 3F and 4F; Figure S1A‐F). The HE tests showed the abundant cytoplasmic colorless gaps existed in the hepatocytes around the Glisson's capsules, but not the central vein (Figure S1D‐F). It is therefore suggested that the specific gaps of the cytoplasm of the hepatocytes is where the immunoreaction occurred by the IHCs‐N for OS or for OC. Furthermore, in the **IHCs‐N for OS**, specific staining was also noticed on the luminal surface of the bile capillaries of all parts of the lobules, continuing on the luminal surface of the intercalated portions and interlobular bile duct cells, while the nuclei of the hepatocytes were not stained (Figure 3B and C). In the **IHCs‐N for OC**, however, no staining occurred on the surface of any lumina leading to the bile flow (Figure 4B and C). The **IHC‐M or ‐S for OS** produced immunoreaction in the nuclei of the hepatocytes, in the Kupffer cells weakly stained, in the unknown large cells strongly stained in the sinusoids near the interlobular triad, and in the endothelium cells of the hepatic sinusoids (Figure 3D). More importantly, the strongly stained CSGs of the two types, the predominant one lining the bile capillaries and the other of a very few CSGs occurred in the weakly stained hepatocytes (Figure 3E and F), together with the strongly stained interlobular bile duct (Figure 3F). The **IHC‐M or ‐S for OC** produced moderate staining for OC in the hepatocytes but without CSGs (Figure 4D and E), and strong staining in the interlobular bile duct cells (Figure 4D and E). At 3‐h post administration, immunoreaction for OS or OC was almost the same but with a little lower intensity than at 1‐h (data not shown). At 24‐h all the IHC‐N, ‐M, and S for OS or for OC no longer produced immunoreaction in the liver specimens (data not shown). The controls, including the absorption control and the control liver from rats not administered with OS, all revealed no staining at all (Figures 3G and H and 4G and H).

### 3.3 Immunoelectron microscopy (IEM) for OS or OC in liver

**FIGURE 5:**
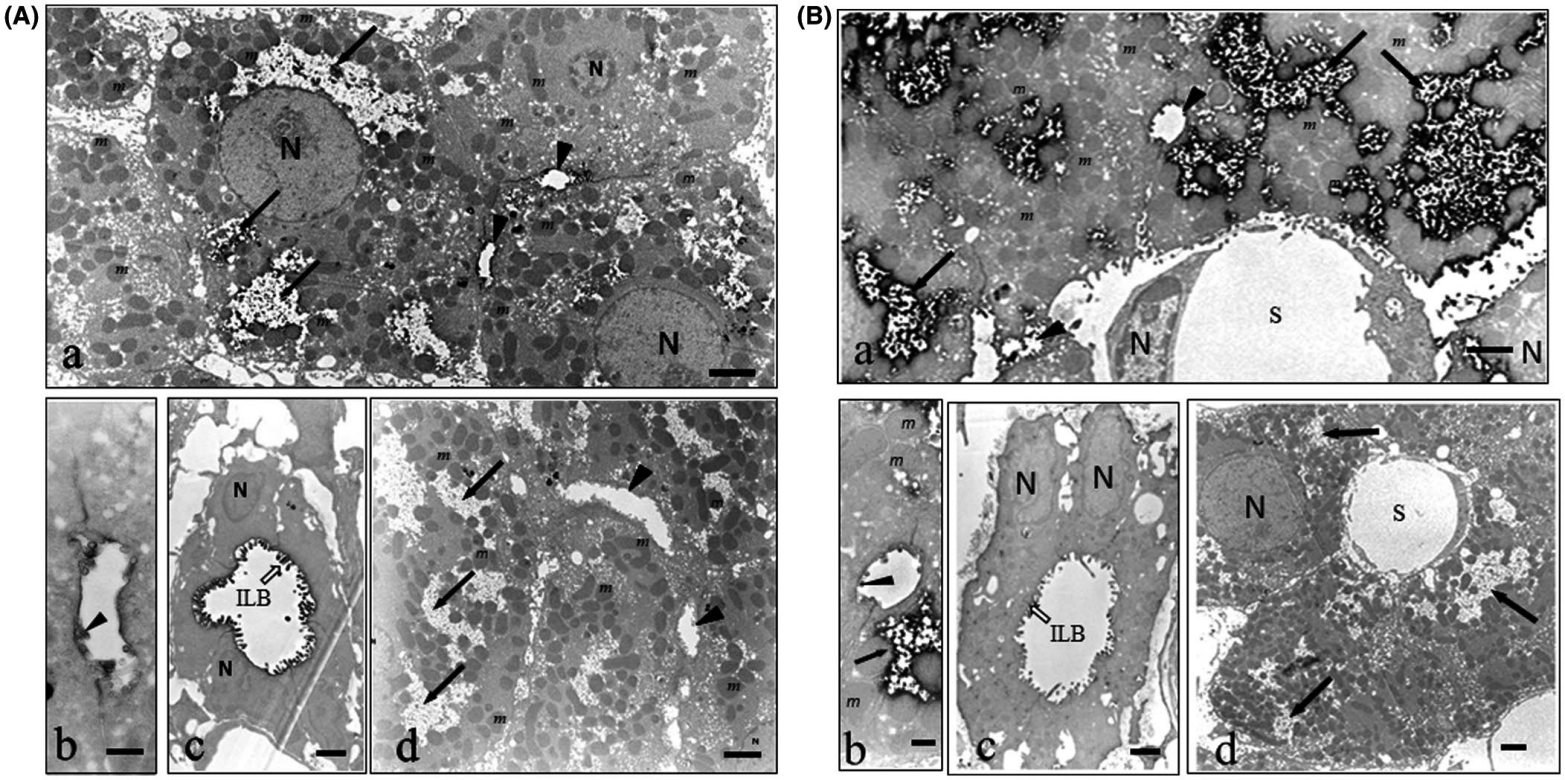
(Aa‐d) Immunoelectron micrographs for OS in the liver of OS‐administered rats. Lower (a, d) and higher magnification (b, c): a‐c Immunoreactivity occurred in the broad fields of the cytoplasm (arrows) peripheral to the nuclei, in the microvilli (arrowhead) of hepatocytes and in the microvilli (open arrow) of the interlobular bile duct cells. (d) Absorption control: The staining was completely abolished by absorption of the AOS‐96 mAb with OS‐GA‐BSA (100 µg/mL). It was clear that the broad field had lower electron density free from any of the subcellular organelles localizing in the periphery of hepatocytes. N nuclei; *m* mitochondria; ILB interlobular bile duct. *Bars* a, d** = **2 µm; b = 500 nm; c = 1 µm. (Ba‐d) Immunoelectron micrographs for OC in the liver of OS‐administered rats. Lower (a, d) and higher magnification (b, c): a‐c Immunoreactivity only occurred in the broad fields of the cytoplasm (arrows), but not in the microvilli (arrowhead) of hepatocytes nor in the microvilli (open arrow) of the interlobular bile duct cells. (d) Staining was completely abolished by absorption of the AOC‐160 mAb with OC‐GA‐BSA (100 µg/mL). N nuclei; *m* mitochondria; *S* sinusoids; ILB interlobular bile duct. *Bars* a, d = 2 µm; b = 500 nm; c = 1 µm

The IEM was developed essentially by the same procedure as the IHC but using 50 µm Microslicer sections of the liver, which, however, had not been pretreated with protease. In the liver specimens at 1‐h post administration, the IEM for OS showed that the immunoreactivity for OS was observed as osmium fine particles in the broad fields in the cytoplasm of the hepatocytes (Figure 5Aa) and in the microvilli of the bile capillaries (Figure 5Ab) and interlobular bile duct cells (Figure 5Ac). In the cytoplasm of the hepatocytes of absorption control, the fields showed lower electron density (Figure 5Ad) and most of the subcellular organelles were located adjacent to the plasma membrane of the hepatocytes (Figure 5Ad). No reaction was found in absorption controls in which 100 µg of OS‐GA‐BSA conjugate was added to the solution of the first immunoreaction with the AOS mAb (Figure 5Ad). The IEM for OC showed that the immune‐positive osmium particles for OC were more abundant in the broad fields in the hepatocytes (Figure 5Ba) than those for OS (Figure 5Aa). On the other hand, no or only very slight immunoreaction was observed in the microvilli of the bile capillaries nor interlobular bile ducts (Figure 5Aa‐C). Absorption controls showed no reaction (Figure 5Bd).

### 3.4 Drug uptake in kidney

**FIGURE 6:**
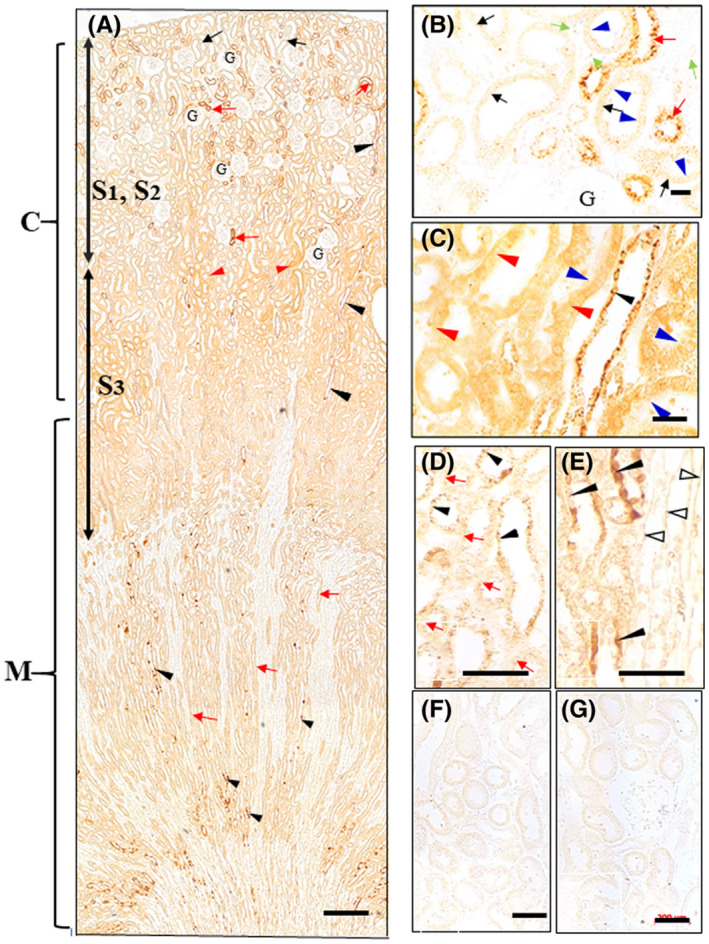
(A‐G) Immunostaining for OS in the kidney of OS‐administered rats. Rats were orally administered OS at 20 mg/kg and then sacrificed 3 hours later. OS IHC was carried out with protease digestion of sections at 0.003% at 30°C for 2 hours (IHC‐S) prior to immunoreaction with AOS‐96 mAb. This severe protease digestion was inevitably needed for the immunostaining, especially in the S1 and S2 segment cells of the proximal tubules. In the process, however, their cell morphology seemed to be extensively damaged, resulting in their microvilli being almost completely disappearing (B). Lower (A) and higher magnification (B‐G): (a) A wide variety of immunostaining was observed in the nephrons composed of the segments or parts, as clearly classified by their specific colors. (B, C) Immunoreactivity was very faint in the microvilli (blue arrowheads), cytoplasm, nuclei of the S1 and S2 segment cells (black arrows), in which, however, the CSGs are still recognized, and moderate staining in the S3 segment cells (red arrowheads) with their damaged villi, which are only very slightly stained (blue arrowheads). The S3 segment was identifiable by the presence of periodic acid‐Schiff‐positive brush borders. Very weakly stained blood capillaries (green arrows) were also observed. Strong immunostaining was exclusively concentrated in the numerous CSGs at the apical sites or all around the nuclei in the cytoplasm of the straight and convoluted distal tubules (red arrows), followed by the collecting ducts (black arrowheads), although the cytoplasm of all the three cell types seem to show only very faint immunostaining. Almost no staining in the glomeruli (G). (D, E) In the area close to the entrance to the inner medulla, many huge or swollen cells, which are strongly stained as a whole cell, are found in the collecting duct cells. The round beads‐like structure of such uncommon cells was also observed in the collecting ducts. Collecting duct cells (black arrowheads), distal tubule cells (red arrows), thin parts of the Henle's loop cells (open arrowheads). (F) Staining was completely abolished by absorption of the AOS‐96 mAb with OS‐GA‐BSA (100 µg/mL). (G) No staining in kidney from control rats. G glomeruli; C cortex; M medulla; *Bars* A = 30 µm; B = 100 µm; C‐F = 200 µm

**FIGURE 7:**
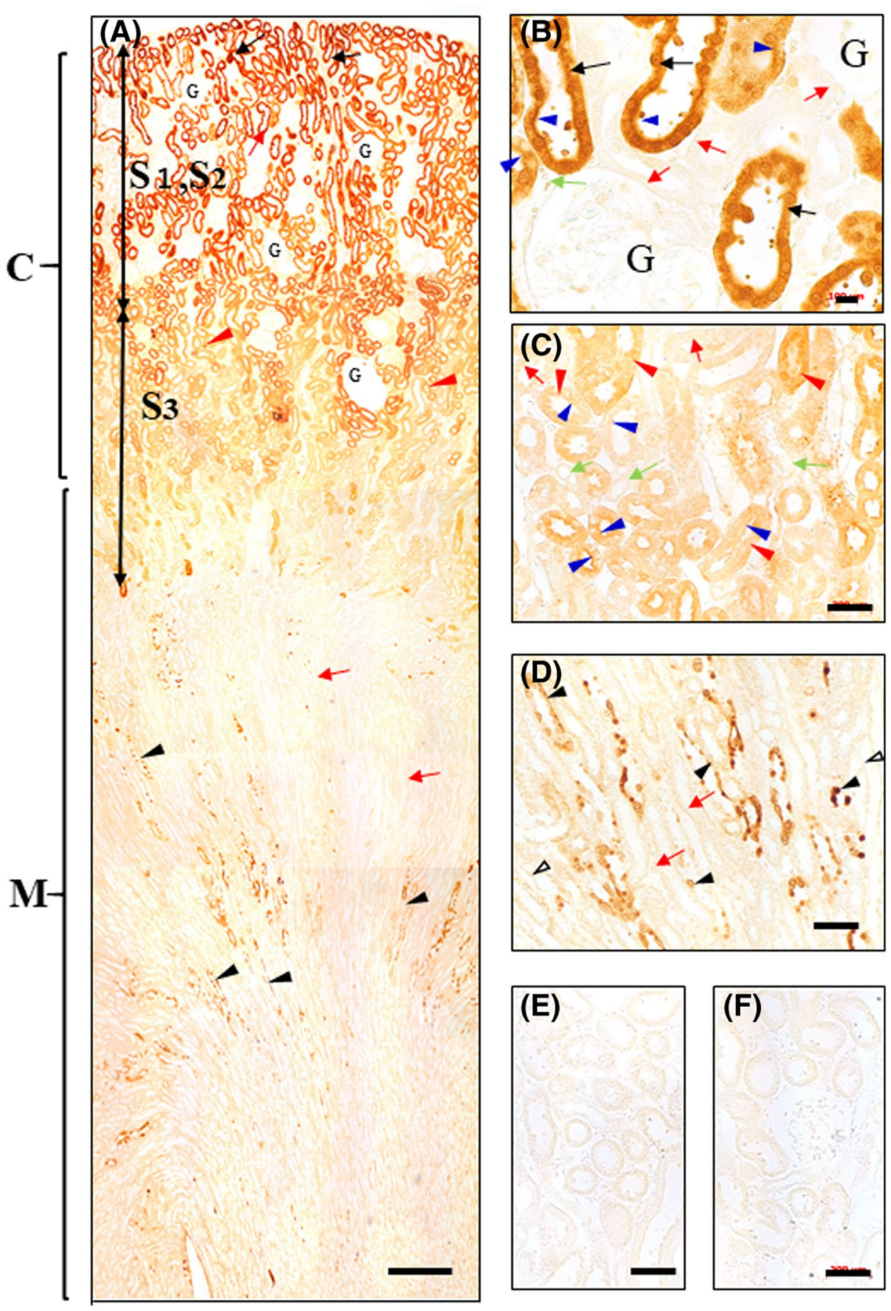
(A‐F) Immunostaining for OC in the kidney of OS‐administered rats. This was carried out in the same manner as that for IHC for OS except using AOC‐160 mAb instead of AOS‐96 mAb, with protease digestion process for 2h (IHC‐S). (A, B) In striking contrast to the faint immunostaining for OS in the S1 or S2 segment of the proximal tubule cells (Figure 6A, B), the very strong staining for OC was observed all over the cells in their segments (black arrows), followed by the less strong staining in the subsequent S3 segment cells of the proximal tubules (red arrowheads). Almost no staining in the glomeruli (G). (C) Immunostaining ranged from very weak to the moderate on the microvilli (blue arrowheads), in the cytoplasm and nuclei of the S3 segment cells (red arrowheads). Almost no or very slight staining in the blood capillary (green arrows). (A, D) Immunostaining almost disappeared suddenly in the renal tubule cells starting from the thin limb of Henle's loop via the straight and convolution distal tubules to the collecting ducts. However, in a manner similar to the findings in IHC‐S for OS (Figure 6A, D), huge or swollen cells, which are strongly stained as a whole cell, or cells in which only the nuclei were strongly stained, scattered in the collecting ducts (black arrowheads). Such uncommon cells were numerous, occurring in the collecting ducts especially around the entrance to the inner medulla. Thin part cells of the Henle's loop (open arrowheads); distal tubule cells (red arrows). (E) Staining was completely abolished by absorption of the AOC‐160 mAb with OC‐GA‐BSA (100 µg/mL). (F) No staining in kidney from control rats. G glomeruli; C cortex; M medulla; *Bars* A = 30 µm; B = 100 µm; C‐F = 200 µm

In the kidney at 3‐h post administration, the IHC‐N for OS or OC produced no staining in any cell types of the nephrons except extraordinarily huge and swollen cells, which were heavily stained as the whole cell, mainly in the collecting ducts especially around the entrance to the inner medulla (data not shown). This staining pattern was quite similar to the results obtained by IHC‐S for OS as well as OC, as described below (Figures 6D and E and 7D and E). The IHC‐S for OS produced no or only very faint staining on the microvilli and occasionally in the nuclei, and moderate staining on abundant CSGs in the S1 and S2 segments of the proximal tubule cells in all which the cytoplasm showed very slight staining (Figure 6A and B). Then, moderate staining occurred in the cytoplasm of the S3 segment cells (Figure 6C). Following almost no staining in the thin parts of the Henle's loop cells, a very strong staining was found almost exclusively in the countless CSGs at the apical sites or all around the nuclei in the cytoplasm of the straight and convoluted distal tubules followed by the collecting ducts, while the cytoplasm of all the three cell types showed only very slight immunoreaction (Figure 6A‐E). Interestingly, huge or swollen cells scattered mainly in the collecting ducts and occasionally in the convoluted distal tubules, as described earlier (Figure 6D and E). Absorption controls and secondary label control showed no reaction in any case (Figure 6F and G). By 24 hour post‐administration slight staining persisted on the CSGs in some collecting ducts, but none in all the other cell types of the renal tubules, as well as in the glomeruli (data not shown).

The IHC‐S for OC clearly showed no CSG in any cell types of the kidney (Figure 7A‐D). No or only a very slight staining for OC distributed in some cells of the glomeruli and the capillaries (Figure 7B). On the other hand, surprisingly, in striking contrast to the OS staining (Figure 6A and B), very strong staining for OC occurred in the microvilli, cytoplasm, and nuclei of the S1 and S2 segment cells of the proximal tubules (Figure 7A‐C). Following weak or moderate staining in the S3 segments, almost no staining suddenly appeared in the thin Henle's loops followed by the distal tubules and collecting ducts. In the similar distribution patterns to those for OS staining (Figure 6D and E), strongly stained huge or swollen cells were found mainly in the collecting ducts and occasionally in the distal tubules, especially near the entrance to the inner medulla (Figure 7D). Absorption controls and secondary label control showed no reaction in any case (Figure 7E and F).

## 4 DISCUSSION

Recently drug delivery studies have extensively advanced, developing controlled release formulations, due to overcoming physicochemical properties of drugs, but have struggled with problems of biological barriers, in which certain transporter systems are closely associated.^49^ However, no fundamental study on the correlation of drug accumulation in animal bodies with such transporters is available, probably because of a lack of a reasonably easy assay procedure for drugs.

We have now prepared and characterized the two mAbs AOS‐96 and AOC‐160 to the conjugates OS‐GA‐BSA and OC‐GA‐BSA, respectively, using GA in the two antigen conjugate preparations. The AOS‐96 and AOC‐160 mAbs were demonstrated to be monospecific for OS and OC with no cross‐reaction with OC and OS, respectively, and unrelated compounds by the inhibition and binding ELISAs (Figure 1Ba, b and Ca, b). No antibody activity against the carrier BSA or GA‐BSA was evident, demonstrating a lack of recognition. These results strongly suggest that the AOS‐96 and AOC‐160 mAbs recognize not only the OS and OC molecules, respectively, but also, in part, carrier protein‐conjugation site(s) of GA, and that both of these mAbs can precisely distinguish the differences in the terminal structures of the ethyl ester group of OS and hydrolyzed carboxylate group of OC, respectively. Such strict antibody specificity of these two mAbs is not surprising, because it is known that antibody is generally produced the best recognizing the farthest chemical group(s) of antigen structure from the site conjugating a carrier protein (BSA).^50^ Furthermore, the fact that each of the mAbs recognized OS or OC conjugated to BSA with GA suggests that both the mAbs could be useful for IHCs for drug uptakes of OS and OC in GA‐fixed tissues.

IHC: In the intestine, the IHC‐S for OS or OC demonstrated that at 3 hour post administration, OS highly distributed in the whole cell of the absorptive epithelia in which strong immunoreaction could be seen in their microvilli, nuclei and CSGs (Figure 2A‐D). OC did similarly so in the epithelia including their microvilli, but without CSGs (Figure 2G‐I). The CSGs were always observed in any cell types including the neurons of the submucous and myenteric plexuses and even the muscular cells in the intestine by the IHC‐S for OS (Figure 2C), but never by the IHC‐S for OC (Figure 2F‐I). In principle, intracellular drug concentrations may reflect the concerted function of the drug cellular influx and efflux, which might be largely influenced by the transporters expressed in the certain cell sites active in absorption, excretion, and transport, and may also reflect the metabolism. OS is actively transported in the epithelia in a mode of transcellular transport, possibly, in parts, in concert with OS absorption in a mode of passive diffusion and, inversely, OS exclusion from P‐gp, since it is, in general, believed that prodrug is absorbed in the former mode, and P‐gp is highly expressed on the microvilli of the epithelia.^19^, ^20^ In addition, it remains unclear if the OS absorption (influx) is correlated with PEPT1 expressed on the microvilli of the intestinal epithelia, because it is under debate if OS is or not the substrate for PEPT1.^30^, ^31^ The high OC levels in the epithelia might imply that the OS uptaken from the intestinal lumina is cleaved to OC within the cells, possibly by the enzyme carboxylesterase 1 (CES1) of the main enzyme involved in intestinal first‐pass hydrolysis,^51^ and persists at high OC levels in the epithelia. This seems more probable than that the high OC levels originate from the circulation bearing OC, although it has been reported that in rats, OS is hydrolyzed by CES1 predominantly in the plasm, but scarcely in the intestine.^52^ Also, the differences in OS levels in the epithelia and in the lamina propria scattering the free cells seemed smaller than those in OC levels (compare Figure 2A and B to F and G, respectively). This suggests that OS more easily penetrates the connective tissues through the basement membrane out of the epithelia, flowing into the blood capillary, during the course where OS more significantly influenced the free cells (lymphocytes, macrophages, plasma cells etc) in the lamina propria.

Liver: The IHCs‐N showed OS distributed on the surface of the lumina ranging from the bile capillaries to the interlobular bile ducts (Figure 3B and C), but OC did not (Figure 4B and C). The IHCs‐S showed OS localized in the lines along the bile capillaries leading to the interlobular bile ducts (Figure 3E‐G), but OC did not do so (Figure 4E). These findings show that OS is excreted via the bile capillary to the bile flows, but OC is virtually not. The bile capillaries’ membrane of the hepatocytes are specific sites, where P‐gp is highly expressed^22^, ^53^, ^54^ strongly suggesting that OS is actually and actively excreted at these sites, possibly through P‐gp, since OS is among substrates for P‐gp.^21^ Previously, we have observed the same bile flow pattern in the IHC for doxorubicin,^32^ the amphiphilic cationic drug known as the substrate for P‐gp.^55^, ^56^ Therefore, it might suggest that drugs classified into a substrate family member for P‐gp are excreted through the bile. It is not yet known what the beautiful staining spots lining the bile capillary were made of (Figure 3E and F), but it is possible that OS released from the bile capillaries was covalently bound via the fixative GA with some proteins occurring in situ, for example, the P‐gp. On the other hand, it is thought that OC might be exported by another excretion path, namely, to the sinusoidal capillary of the blood stream via the basolateral membrane of the hepatocytes expressing Mrp4 for OC,^1^, ^57^ thus suggesting that the OC efflux might be mediated by the Mrp4. Surely, the liver specimens of 12‐h post administration left no OC in the hepatocytes, as observed by the IHCs for OC (data not shown). Thus, despite the indispensability of the direct demonstration of drug‐transporter(s) interaction, this might be the first example of transporter(s) dividing an excretion of the drugs into the bile and kidney. In the interlobular bile duct cells, OC was detected by the IHC‐M or IHC‐S for OC better than by the IHC‐N for OC (compare Figure 4 D and E to B), while it was not detected in the bile flows from the bile capillary (Figure 4E). Perhaps OC predominately originates from circulating OC in the liver artery branches winding the interlobular bile ducts.^58^ The IHC‐M for OS showed a few large cells with high OS levels, lying in the sinusoids (Figure 3D). Thus, the cells might be the Kupffer cells, which show active phagocytosis, swallowing large amounts of OS and growing large in size, which was in contrast to that of the IHC‐M for OC, which showed ordinary small Kupffer cells with lower OC levels (Figure 4D). The results of the IEMs for OS and OC agree well with those of the respective light microscopic IHCs‐N for OS and OC. The IEM showed that immunoreaction for OS or OC occurred in the specific wide fields in the cytoplasm (Figure 5Aa and Ba), which is responsible for the colorless gaps under the light microscopy (Figures 3F and 4F), holding the lower electron density (Figure 5Ad and Bd) and placing the other cell organelles near the cell membranes (Figure 5Aa and Ba). This may be where the immunoreaction by IHC‐N for OS or OC occurred (Figures 3A‐C and 4A‐C). It might, therefore, be reasoned that the drugs in these parts did not undergo any masking effect, probably due to the fact that they were free from the other cell organelles, and were easily washed away in the protease digestion process of the IHCs‐S for both drugs. Also, the space of the specific gaps seemed rather inflexible, since no significant morphological changes occurred in time‐different specimens of the livers from OS‐administered rats or controls (Figure S1A‐F).

Kidney: At the 3‐h post‐administration, OS distributed only slightly in the cytoplasm of the S1 and S2 proximal tubules, except for the S3 segment with some OS levels (Figure 6A‐C). This continued in the cytoplasm of the consecutive renal tubules from the thin limb of Henle's loop to the collecting ducts, while OS were highly concentrated only in the CSGs in those cells (Figure 6B‐E). Taking into account that P‐gp are expressed on the microvilli of the proximal tubules and also the Mdr‐1b mRNA in almost entire nephrons,^59^ it might be assumed that OS is sooner excreted by the efflux P‐gp than it takes time to OS is metabolized in the CSGs.^60^ Meanwhile, it remains unclear if PEPT1 is involved in re‐absorption of OS.^61^ The best way to elucidate the correlation of transporters with drug disposition is to histochemically demonstrate their co‐localization, but it is impossible, because different fixatives for diffusible drug and protein are inevitably needed for each other. The CSG might appear responsible for the lysosome in the tubules, which have been demonstrated by gentamicin autoradiography,^62^ since in our IHCs for gentamicin,^36^ vancomycin^46^ and OS all the same types of granules as those of the autoradiography were observed. This seems quite reasonable since the lysosome is the subcellular organelle active for the phagocytosis. Moreover the CSGs were observed in any cell types in the different tissues. This was first demonstrated by the present IHC in which the surfactant saponin was contained in the secondary antibody solution, as well, expecting the better immunoreaction with drug in situ*.* ^63^ All data together with the chemical structure of OS suggest that OS, but not OC, is among the “Lysosomotropic" cationic amphiphilic drugs.^64^

OC highly distributed in the S1 and S2 proximal tubules, and then immediately disappeared in entire cells from the thin limb of Henle's loop to the collecting ducts: The transporters for OC, OAT1^24^, ^65^, ^66^ and OAT3^23^, ^66^ on almost entire renal tubules^66^, ^67^ and Mrp4^1^ on the proximal tubules are known to be involved in influx^67^ and efflux pumps,^25^, ^27^ respectively. It is, however, impossible at present to reasonably explain the contrasting phenomena of OC levels in the proximal tubules and in all the other cell types of the nephrons, suggesting that there are other factors and/or unknown transporters.

Additionally, IHCs‐N as well as IHCs‐S for OS or OC showed that both the drugs brought about all the similar phenomena in the kidney that huge or swollen cells with the extraordinary high levels of each of the drugs localized mainly in the collecting ducts, especially around the entrance to the inner medulla (Figures 6D and E and 7D), suggesting that most of these cells might be damaged. Also, the number of such unusual cells for OS or OC seemed comparable to each other (Figure 6A and 7A). This might mean that both drugs almost equally possess the renal toxicity in rats. The collecting ducts might be more fragile for the drugs than any other renal tubules since we have previously observed the similar phenomena in the IHCs for gentamicin,^36^ amoxicillin,^33^ peplomycin,^37^ and vancomycin,^46^ and others.^68^, ^69^ This might possibly happen because ca. 5‐times larger dosage than that for the clinical trials per a day was administered rats in this study.^3^

## 5 DATA REPOSITORY LINK

The authors declare that at the time of this study was published, data repository link was not available for the authors at Sojo University or Jikei University.

## ETHICS STATEMENT

Ethics approval for this study was obtained from the Sojo University Animal Experiment Ethics Committee.

## DISCLOSURE

The authors have no conflict of interest to declare.

## AUTHOR CONTRIBUTIONS

Fujiwara *participated in research design.* Fujiwara, Yamamoto, and Saita *conducted experiments*. Fujiwara, Yamamoto, Saita, and Matsufuji *performed data analysis*. Fujiwara *wrote the manuscript*.

## Supporting information

Supplementary Material
