# Supplementary figures and images for: Metabolism and disposition of oseltamivir (OS) in rats, determined by immunohistochemistry with monospecific antibody for OS or its active metabolite oseltamivir carboxylate (OC): A possibility of transporters dividing the drugs’ excretion into the bile and kidney
Source: Pharmacol Res Perspect. 2020 Jun 2;8(3):e00597. doi: 10.1002/prp2.597 (PMC7266928; doi:10.1002/prp2.597)

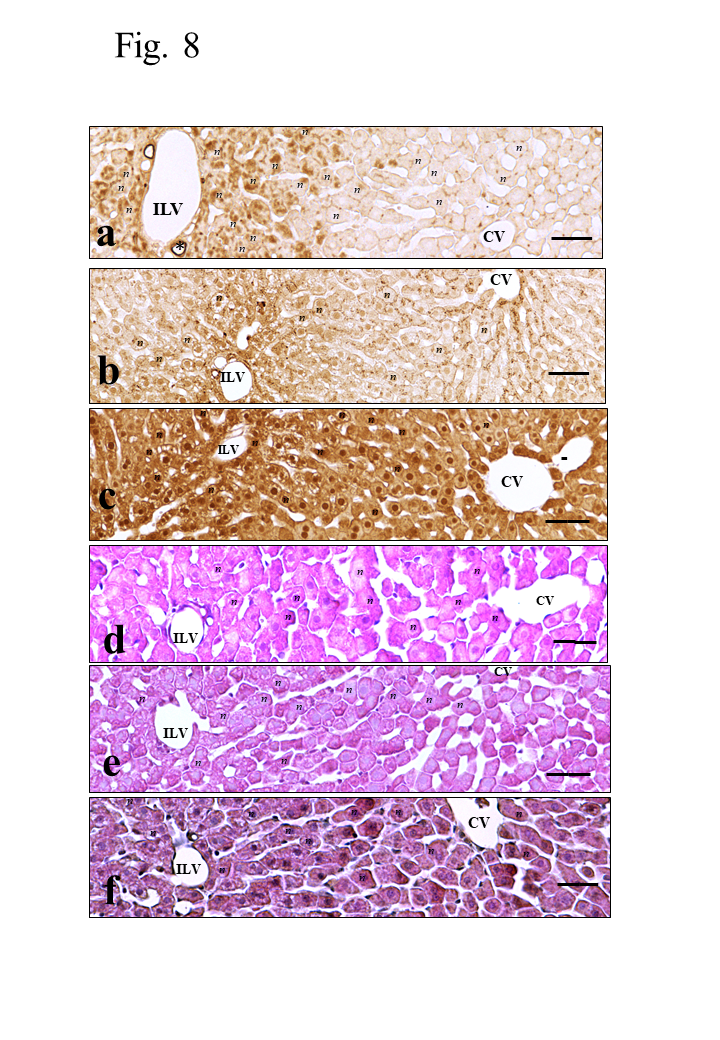

Supplement: Supplementary file 1 — Supplementary Material [file PRP2-8-e00597-s001.TIF]
